# Prenatal exposure to polychlorinated biphenyls (PCBs) and polybrominated diphenyl ethers (PBDEs) may influence birth weight among infants in a Swedish cohort with background exposure: a cross-sectional study

**DOI:** 10.1186/1476-069X-12-44

**Published:** 2013-05-31

**Authors:** Sanna Lignell, Marie Aune, Per Ola Darnerud, Annika Hanberg, Susanna C Larsson, Anders Glynn

**Affiliations:** 1 Risk Benefit Assessment Department, National Food Agency, Box 622, Uppsala SE-751 26, Sweden; 2 Science Department, National Food Agency, Box 622, Uppsala SE-751 26, Sweden; 3 Institute of Environmental Medicine, Karolinska Institutet, Box 210, Stockholm SE-171 77, Sweden

**Keywords:** Birth weight, Breast milk, PBDE, PCB, BMI, Weight gain, Fish consumption

## Abstract

**Background:**

Prenatal exposure to persistent organic pollutants, e.g. polychlorinated biphenyls (PCBs) and polybrominated diphenyl ethers (PBDEs) has been suggested to negatively affect birth weight although epidemiological evidence is still inconclusive. We investigated if prenatal exposure to PCBs and PBDEs is related to birth weight in a Swedish population with background exposure.

**Methods:**

Breast milk was sampled during the third week after delivery from first-time mothers in Uppsala county, Sweden 1996–2010 (POPUP cohort) (N = 413). Samples were analysed for di-*ortho* PCBs (CB-138, 153, 180) and tetra- to hexa- brominated PBDEs (BDE-47, 99, 100, 153). Simple and multiple linear regression models were used to investigate associations between lipid-adjusted, ln-transformed PCB and PBDE concentrations, and birth weight. Covariates included in the multivariate regression model were PCB and PBDE exposure, maternal age, pre-pregnancy BMI, weight gain during pregnancy, education, smoking, gender of the infant and gestational length. The effect of including fish consumption was also investigated.

**Results:**

In the multivariate model, prenatal exposure to di-*ortho* PCBs was significantly associated with increased birth weight (β = 137; p = 0.02). The result did not change when gestational length was added to the model. An inverse association between PBDE(4) (sum of BDE-47, -99, -100 and −153) and birth weight was observed in the multivariate model including gestational length (β = −106; p = 0.04). Maternal pre-pregnancy BMI and weight gain during pregnancy were important confounders of the association between di-*ortho* PCBs and birth weight. The associations were not alleviated after adjustment for fish consumption, a major source of PCB and PBDE exposure. The observed associations were stronger for boys than for girls.

**Conclusions:**

Our results indicate that prenatal exposure to di-*ortho* PCBs and PBDE(4) may influence birth weight in different directions, i.e. PCB exposure was associated with higher birth weight and PBDE exposure with lower birth weight. Maternal pre-pregnancy BMI and weight gain during pregnancy were important confounders that may hide positive association between di-*ortho* PCB exposure and birth weight if they are not included in the statistical model. We speculate that even small PCB- and PBDE-induced shifts in the distribution of birth weight may influence future public health in populations with background exposure.

## Background

Toxic persistent organic pollutants (POPs), such as polychlorinated biphenyls (PCBs), polychlorinated dibenzo-*p*-dioxins (PCDDs), polychlorinated dibenzofurans (PCDFs), *p,p’*-DDE and polybrominated diphenyl ethers (PBDEs), contaminate the environment due to human activities. Among other potential effects, PCBs and PCDD/Fs are suggested to cause reduced birth weight [1-4] and there are also studies showing that PCBs and DDE may affect growth and body size during the first years of life [3,5,6]. Among the few studies on associations between PBDE exposure and birth weight, some have shown inverse relationships between prenatal exposure and birth weight [7,8]. Both high and low birth weight is related to overweight later in life [9-11] and currently the role of early exposure to endocrine disrupting POPs on later development of obesity is debated [12,13]. Possible effects of prenatal POP exposure on birth weight are consequently important to study.

The aim of this study was to investigate if prenatal exposures to di-*ortho* PCBs and tetra- to hexa brominated PBDEs are related to birth weight in a Swedish population of first-time mothers with background exposure. The level of di-*ortho* PCBs is a good marker for dioxin-like PCBs and PCDD/Fs in Sweden [14], and the results may consequently be relevant for these substance groups too. We collected information about several possible confounding factors, e.g. maternal age, maternal pre-pregnancy BMI (body mass index), weight gain during pregnancy, education, smoking and fish consumption. The inclusion of maternal pre-pregnancy BMI and weight gain in the statistical model is of special interest since BMI and weight gain are positively associated with birth weight and negatively associated with levels of PCBs in breast milk [15]. Fish consumption is also a possible confounder. Fish is a major source of exposure to POPs, including PBDEs, in the Swedish population [16], and at the same time, fish/seafood consumption has previously been positively associated with birth weight [17-19]. Furthermore, we assessed whether there are sex differences in the associations between PCB and PBDE exposure and birth weight as indicated in a few studies [20-22].

In this article we report crude and adjusted associations between prenatal exposure to di-*ortho* PCBs and tetra- to hexa brominated PBDE and birth weight in a cohort of Swedish first-time mothers. Prenatal exposure was estimated by levels in breast milk sampled three weeks after delivery.

## Methods

### Study population, sampling and data collection

The POPUP study (Persistent Organic Pollutants in Uppsala Primiparas) started in 1996, and up to 2010 we recruited 526 first-time mothers born in Nordic countries and living in Uppsala County. In 1996 to 1999, participants were randomly recruited in late pregnancy and donated a blood sample in gestational week 32–34 (N = 316) [23]. 203 of these women continued to participate in the study after delivery and donated a breast milk sample [14]. In 2000–2010, women were recruited after delivery and were asked to donate breast milk (N = 210) [24]. The milk sampling was conducted at home during the third week after delivery [24].

Data on maternal and child characteristics were obtained from questionnaires or from the Swedish Medical Birth Register at the National Board of Health and Welfare [15,23]. 118 women (29%) did not permit the project to collect data from registers, and a mixture of questionnaire and register data was therefore used for some variables (maternal weight and length before pregnancy, weight gain during pregnancy, diabetes, hypertensive disorders, preeclampsia, birth weight and sex of the child). For individuals with data on maternal weight and length before pregnancy, weight at delivery, and birth weight, available from both questionnaire and register (N = 257-295), the correlations between data sources were very strong (Pearson correlation coefficients 0.97-0.99). Data on fish consumption during the year before pregnancy was obtained from a food frequency questionnaire that was answered by the participants when the child was approximately 6 weeks old [15]. The study was approved by the local ethics committee of the Uppsala region, and participants gave their informed consent prior to the inclusion in the study.

### Chemical analysis

All breast milk samples were analysed for the di-*ortho* substituted PCB congeners PCB 138, PCB 153 and PCB 180 (N = 413), and a randomly selected number of samples (N = 364) for four PBDE-congeners (BDE-47, BDE-99, BDE-100, BDE-153). The analyses were performed at the Swedish National Food Agency (NFA), and have been described earlier [24]. After extraction and separation, the final analyses of PCBs were performed on a gas chromatograph with dual capillary columns of different polarity and dual electron-capture detectors. In samples from 2008 and 2010, di-*ortho* PCBs were analysed by gas chromatography coupled to high-resolution mass spectrometry [25]. To compare the two methods for PCB analysis, a calibration study was performed where 20 milk samples were analysed with both methods. The agreement between results was satisfactory [25]. PBDEs were analysed by gas chromatography/low resolution mass spectroscopy/electron capture negative ionization and detected by single ion monitoring technique [24]. The total lipid content of each breast milk sample was determined gravimetrically. A number of control and blank samples were inserted in each batch of samples to verify the accuracy and precision of the measurements. The laboratory is accredited and has successfully participated in several international proficiency tests.

### Data analysis

Lipid-adjusted breast milk levels of di-*ortho* PCBs and PBDEs were used to estimate prenatal exposure since these levels give a good estimate of the maternal body burden during pregnancy, and consequently the exposure of the fetus during pregnancy [26,27]. For samples from 1996–2008 (N = 305) with PBDE concentrations below the limit of quantification (LOQ), half of LOQ was taken as an estimated value. LOQs for the PBDE congeners were 0.29-1.2 ng/g lipid (BDE-47), 0.12-0.88 ng/g lipid (BDE-99), 0.05-0.64 ng/g lipid (BDE-100) and 0.23-0.34 ng/g lipid (BDE-153), respectively. In samples from 2009 and 2010 (N = 59), BDE-47, BDE-99 and BDE-100 levels between the limit of detection (LOD) and LOQ were reported by the laboratory (N = 30, 54 and 7 respectively). To improve the statistical power, these levels (corrected for levels in blank samples) were used instead of half of LOQ in the statistical analyses. One sample had levels of BDE-99 below LOD.

Correlation analysis (Pearson) and one-way analysis of variance (ANOVA) were used to investigate univariate associations between birth weight and individual characteristics of the mothers.

We used simple and multiple linear regression models (MINITAB 15® Statistical Software for Windows) to analyse associations between prenatal exposure to di-*ortho* PCBs (sum of PCB 138, 153 and 180) and PBDE(4) (sum of BDE-47, BDE-99, BDE-100 and BDE-153) and birth weight. Logarithmically transformed exposure data were used since the distribution of data closely followed a log-normal distribution. Birth weight was normally distributed in the study population.

In the crude analyses, the associations between di-*ortho* PCBs or PBDE(4) and birth weight were investigated in separate models. In the adjusted model, di-*ortho* PCBs and PBDE(4) were included simultaneously. In addition, covariates included in the adjusted model were maternal age, pre-pregnancy BMI, weight gain during pregnancy, education, smoking during pregnancy and sex of the child. These variables have all been associated with birth weight [28-33]. Covariates were included as continuous variables or categorized according to Table 1. Gestational length, a major predictor of birth weight, was added to the model in a second step to investigate if observed associations between exposure and birth weight were mediated by effects on gestational length. The influence of adding fish consumption [19] to the statistical model was also investigated. To study possible sex differences in the relation of birth weight to exposure, all models were ran for male and female infants separately. Since data on PBDE(4) and gestational length was missing for many participants, analyses restricted to individuals with complete datasets were also performed to enable comparisons between models. We performed correlation analysis between the independent variables in the models and also used variance inflation factors (VIFs) to check for collinearity in the adjusted regression models. The fits of the regression models were checked by plotting the residuals around the fitted line. The robustness of the results was evaluated by a sensitivity analysis where outliers with a standardized residual ≥3 were excluded from the regression models. A p-value ≤0.05 was considered statistically significant in all statistical tests.

**Table 1:** Characteristics of the first-time mothers and infants included in the study

**Characteristic**	**N**	**(%)**	**Mean (Range)**
Age (years)	413		28.8 (17–41)
Sampling year	413		
1996-1999	203	(49%)	
2000-2003	58	(14%)	
2004-2006	62	(15%)	
2008-2010	90	(22%)	
Pre-pregnancy BMI (body mass index, kg/m^2^)	412		23.4 (15.9-39.7)
Weight gain during pregnancy (%)	411		23 ((−6)-54)
Years of education	389		
≤ 4 years of high school	138	(35%)	
1–3 years of higher education	76	(20%)	
>3 years of higher education	175	(45%)	
Smoking habits during pregnancy	411		
Smoker	53	(13%)	
Non-smoker	302	(73%)	
Former smoker	56	(14%)	
Consumption of fish (g/day)	374		29 (0–134)
Gestational length (days)	295		281 (249–302)
Birth weight of the child (g)	411		3589 (2430–5110)
Sex of the child	411		
Male	218	(53%)	
Female	193	(47%)	

## Results

### Individual characteristics and contaminant levels

Characteristics of the participating women and children are presented in Table 1. Correlation analysis and one-way ANOVA showed that pre-pregnancy BMI, weight gain during pregnancy, gestational length and sex of the child (male versus female infant) were positively associated with birth weight (p≤0.05, results not shown). Smoking during pregnancy, a known risk factor for reduced birth weight [34], was not associated with birth weight, probably because of the relatively small number of cases. Most women categorised as smokers stopped smoking during the first trimester. There was a weak positive but non-significant correlation between fish consumption and birth weight (Pearson correlation coefficient 0.07, p = 0.2) and no significant correlations between birth weight and maternal age or education.

Data on breast milk levels of di-*ortho* PCBs and PBDEs among the women recruited in 1996–2010 are presented in Table 2. Di-*ortho* PCB data was available for all study participants (N = 413) and PBDEs in samples from 364 randomly selected participants from 1996 to 2010. PCB 153 was the di-*ortho* PCB congener with the highest mean concentration and the highest mean level among the PBDEs was shown for BDE-47. The levels of di-*ortho* PCBs were about thirty times higher than the levels of PBDE(4).

**Table 2:** PCB and PBDE levels in breast milk from the mothers in the study

**Substance**	**N**	**Mean (SD**^**a**^**)**	**Median**	**Range**	**N < LOQ**
di-*ortho* PCBs^b^	413	103 (54)	90	15-363	-
PCB 153	413	52 (29)	44	7.0-186	0
PCB 138	413	26 (14)	23	4.5-94	0
PCB 180	413	25 (13)	22	2.6-84	0
PBDE(4)^c^	364	3.1 (2.6)	2.4	0.45-28	-
BDE-47	364	1.7 (1.7)	1.3	<LOQ-16	46
BDE-99	364	0.38 (0.47)	0.26	<LOQ-5.2	124
BDE-100	364	0.34 (0.40)	0.25	<LOQ-5.1	55
BDE-153	364	0.65 (0.50)	0.54	<LOQ-4.7	6

^a^Standard deviation.

^b^Sum of PCB 138, PCB 153 and PCB 180 (ng/g lipid).

^c^Sum of BDE-47, BDE-99, BDE-100 and BDE-153 (ng/g lipid).

### Associations between exposure and birth weight

In the multivariate model, there was a significant positive association between di-*ortho* PCB exposure and birth weight (Table 3). The mean increase in birth weight was 137 g (95% confidence interval: 17–257) for every 1-unit increase in ln-transformed di-*ortho* PCB concentration. This corresponds to a mean difference in birth weight between the 25th (64 ng/g lipid) and 75th (132 ng/g lipid) percentile of di-*ortho* PCB exposure of approximately 100 g. When the analysis was restricted to individuals with data on gestational length, the p-value increased to 0.08, but the β-coefficient was essentially unchanged (Table 3). The association became slightly weaker when outliers were excluded from the analysis (β = 120; p = 0.04). Maternal pre-pregnancy BMI, weight gain during pregnancy and PBDE(4) exposure were identified as the most important confounders. When any of these covariates were excluded from the regression model, the association between di-*ortho* PCB exposure and birth weight became non-significant. Adding gestational length to the multivariate model did not significantly change the result for di-*ortho* PCBs (Table 3). Further inclusion of fish consumption slightly increased the beta-coefficient with 13% (β = 162; p = 0.02). There was no indication of collinearity in any of the models (VIFs <2). When female and male infants were studied separately, the multivariate model showed a significant positive association between di-*ortho* PCB and birth weight in male infants, but not in females (Table 4). This result did not change when the analysis was restricted to individuals with data on gestational length (Table 4).

**Table 3:** **Associations between exposure to di-*ortho *PCBs and PBDEs and birth weight**

	**β**^**a**^	**SE**^**b**^	**p**	**N**
Unadjusted model^c^				
di-*ortho* PCBs^d^	−26	44	0.6	411
di-*ortho* PCBs^d^ - restricted^e^	2	48	1.0	346
PBDE(4)^f^	−49	40	0.2	362
PBDE(4)^f^ – restricted^e^	−47	42	0.3	346
Multivariate model^g^				
di-*ortho* PCBs^d^	137	61	0.02*	346
di-*ortho* PCBs^d^ - restricted^h^	131	74	0.08	254
PBDE(4)^f^	−54	46	0.2	346
PBDE(4)^f^ – restricted^h^	−117	59	0.05*	254
Multivariate model including gestational length^i^				
di-*ortho* PCBs^d^	143	65	0.03*	254
PBDE(4)^f^	−106	52	0.04*	254

^a^Regression coefficient, i.e. change in birth weight per 1-unit increase in ln-transformed contaminant concentration in breast milk.

^b^Standard error.

^c^Di-*ortho* PCB and PBDE(4) were included in separate models.

^d^Sum of PCB 138, PCB 153 and PCB 180.

^e^Regression analysis restricted to individuals with data on all covariates included in the multivariate model.

^f^Sum of BDE-47, BDE-99, BDE-100 and BDE-153.

^g^Di-*ortho* PCB and PBDE(4) were included in the same model. Other covariates included were age of the mother, pre-pregnancy BMI, weight gain during pregnancy, education, smoking and infant sex.

^h^Multivariate regression analysis restricted to individuals with data on gestational length.

^i^Gestational length was added to the covariates in the multivariate model.

*p≤0.05.

**Table 4:** Associations between exposure to PCBs and PBDEs and birth weight among female/male infants

	**Female infants**				**Male infants**			
	**β**^**a**^	**SE**^**b**^	**p**	**N**	**β**^**a**^	**SE**^**b**^	**p**	**N**
Multivariate model^c^								
di-*ortho* PCBs^d^	42	99	0.7	161	200	77	0.01*	185
di-*ortho* PCBs^d^ – restricted^e^	88	136	1.0	110	189	88	0.03*	144
PBDE(4)^f^	32	70	0.7	161	−126	62	0.04*	185
PBDE(4)^f^ – restricted^e^	−20	104	0.8	110	−162	73	0.03*	144
Multivariate model including gestational length^g^								
di-*ortho* PCBs^d^	149	123	0.2	110	141	77	0.07	144
PBDE(4)^f^	−47	93	0.6	110	−139	64	0.03*	144

^a^Regression coefficient, i.e. change in birth weight per 1-unit increase in ln-transformed contaminant concentration in breast milk.

^b^Standard error.

^c^Di-*ortho* PCB and PBDE(4) were included in the same model. Other covariates included were age of the mother, pre-pregnancy BMI, weight gain during pregnancy, education, smoking and infant sex.

^d^Sum of PCB 138, PCB 153 and PCB 180.

^e^Multivariate regression analysis restricted to individuals with data on gestational length.

^f^Sum of BDE-47, BDE-99, BDE-100 and BDE-153.

^g^Gestational length was added to the covariates in the multivariate model.

*p≤0.05.

Although not significant, the association between PBDE(4) and birth weight was inverse in the unadjusted as well as in the multivariate model (Table 3). When the multivariate model was restricted to individuals with data on gestational length, the β-coefficient changed markedly from −54 to −117 and the association became significant (Table 3). Adding gestational length to the model did not further change the result (β = −106). The result was similar when outliers were excluded from the analysis (β = −116; p = 0.02). If di-*ortho* PCB was excluded from the statistical model, the association became non-significant and β changed from −106 to −47. The association between PBDE(4) and birth weight became stronger when fish consumption was included in the multivariate model, β decreased with 29% from −106 to −137 (p = 0.01). When female and male infants were studied separately, the multivariate models showed significant inverse associations between PBDE(4) and birth weight in male infants, but not in females (Table 4). Excluding individuals with missing data on gestational length from the multivariate model did not change this conclusion although the observed association in male infants became stronger (β changed from −126 to −162) (Table 4). There was no indication of collinearity in any of the models (VIFs <2).

## Discussion

Our study showed a weak but significant positive association between background prenatal exposure to di-*ortho* PCBs and birth weight. A shift towards higher birth weights at higher PCB exposures may be non-beneficial since positive associations between birth weight and risk of obesity later in life have been demonstrated [11,35]. In contrast to our results, accidental exposures to very high levels of PCDD/Fs and PCBs have been shown to decrease birth weight [4,36]. Among other studies of associations between background exposure to PCBs and birth weight, some report inverse associations [1-3,21,37-41], while other report no significant associations [42-48]. One study presenting a positive association between PCB exposure and birth weight [49] is uncertain since the exposure assessment was based on consumption of contaminated fish rather than on analysed PCB levels. The inconsistent results between studies may for example be due to differences in study design, exposure levels, congener profiles, definition of PCB exposure (sum of different number of congeners or TEQs) and statistical analyses. Generally, the PCB exposure levels in studies showing inverse associations between PCB exposure and birth weight were higher than in the POPUP cohort. For example, the mean cord serum PCB 153 level in the cohorts included in the meta-analysis by Govarts et al. [37] was 180 ng/L. Using the same formulas as Govarts et al., this can be converted to a breast milk level of approximately 150 ng/g fat, which is about three times the mean PCB 153 level in the POPUP cohort. We speculate that a non-monotonic dose-effect curve may explain some of the discrepancies between studies, with a positive low-dose effect of di-*ortho* PCBs on birth weight and a negative effect at higher exposure levels. For endocrine disrupting chemicals, different effects at low and high doses are currently discussed [50].

In our study, the positive association between di-*ortho* PCB exposure and birth weight in the multivariate model was observed only after adjustment for maternal pre-pregnancy BMI, weight gain during pregnancy and concurrent exposure to PBDE(4). Increased pre-pregnancy BMI and weight gain are both associated with increased birth weight as well as with decreased levels of PCBs in breast milk and maternal blood [15,23], and are consequently important confounders. The correlation between pre-pregnancy BMI and weight gain was significant but weak in the POPUP-cohort (Pearson correlation coefficient −0.3, p < 0.001) and the VIFs in the multivariate model did not indicate any problems with collinearity in the regression analyses.

Many studies like ours adjust their results for maternal pre-pregnancy BMI, but among studies showing inverse associations or no associations between birth weight and PCB exposure, Lopez-Espinosa et al. [46] is the only one we could find that adjust for both BMI and weight gain. In a recent meta-analysis [37], adjusting for both BMI and weight gain was possible in four of the twelve investigated cohorts. However, the authors conclude that this adjustment did not change the risk estimates considerably. Nevertheless, we found that weight gain is an important covariate, and it is possible that inverse associations between birth weight and PCBs in some of the other studies are at least partly confounded by maternal weight gain. This should be considered in future studies.

In addition to maternal pre-pregnancy BMI and weight gain, concurrent exposure to PBDE(4) was an important covariate in the analysis of associations between di-*ortho* PCB exposure and birth weight. It could be speculated that PCBs and PBDEs influences birth weight via biochemical pathways in such way that exposure to PBDE(4) may to some extent mask the association between di-*ortho* PCBs and birth weight and vice versa. When the multivariate regression analysis was restricted to individuals with data on gestational length, the association did not change, although it became non-significant most probably because of a decreased statistical power (Table 3). Adding gestational length to the model did not significantly change the result (Table 3), indicating that the association between di-*ortho* PCB exposure and birth weight was mediated by increased fetal growth rather than by increased gestational length.

We can only speculate about a possible mechanism responsible for the observed positive association between birth weight and di-*ortho* PCBs, if causal. Maternal estrogen levels during pregnancy have been linked to increased birth weight [51,52], and estrogenic as well as anti-estrogenic effects of PCBs have been shown in different *in vitro* and *in vivo* bioassays [53-56]. It is possible that di-*ortho* PCBs alone are not responsible for the positive association with birth weight. In addition to di-*ortho* PCBs and PBDEs, a selected number of breast milk samples from the POPUP cohort have previously been analysed for non-*ortho* PCB congeners, 2,3,7,8-substituted polychlorinated dibenzo-*p*-dioxins (PCDDs) and polychlorinated dibenzofurans (PCDFs) and for *p,p’*-DDE (N = 242-383) [24]. The correlations between di-*ortho* PCB levels and the levels of non-*ortho* PCB TEQs (toxic equivalents), PCDD/F TEQs and *p,p’*-DDE were strong (Pearson correlation coefficients 0.7-0.9), showing that di-*ortho* PCB is a good marker for exposure to the other compounds. Consequently, it is difficult to separate the influence of different chlorinated compounds on birth weight from each other, and it is possible that di-*ortho* PCB exposure is a proxy for exposure to the total mixture (‘cocktail’).

Our results showed an inverse association between prenatal exposure to PBDE(4) and birth weight. The association was significant in the multivariate regression model including di-*ortho* PCB exposure and gestational length as covariates. A β-coefficient of −106 corresponds to a mean difference in birth weight between the 25th (1.6 ng/g lipid) and 75th (3.5 ng/g lipid) percentile of PBDE(4) exposure of approximately −80 g. The association was similarly significant in the multivariate model (without gestational length), when the analysis was restricted to individuals with data on gestational length (Table 3). This suggests that the observed association was not due to a PBDE influence on gestational length. In female infants, restricted analysis resulted in a shift from a positive to an inverse association (although not significant), and in male infants, the inverse association became stronger (Table 4). These changes in female and male infants contribute to the shift from a non-significant association in the unrestricted analysis on the total cohort to the significantly inverse association in the restricted analysis. It is from our data not possible to draw firm conclusions about the reason behind the difference between the unrestricted and restricted analysis, but it may be due to chance. Nevertheless, both in the unrestricted and restricted analysis we found an inverse association between prenatal PBDE exposure and birth weight among boys, showing that this association was robust to changes in the number of study participants in the two analyses.

There are few other studies on associations between PBDEs and birth weight, and these studies report no [57,58] or inverse associations [7,8,59,60] depending on congeners studied and statistical models used. Some of the studies are very small (N = 12-41) [57-59] and/or only report unadjusted correlations [57,60]. In the study by Foster et al. [7] (N = 97), there was an inverse association between BDE-99 in umbilical cord serum and birth weight. Harley et al. [8] (N = 286) reported significant inverse associations between BDE-47, -99 and −100 in maternal blood serum and birth weight, but the significant associations did not persist when controlling for maternal weight gain.

Despite considerably lower exposure levels in the POPUP cohort than in the studies by Foster et al. [7] and Harley et al. [8], we observed a significant inverse association between PBDE(4) exposure and birth weight when maternal weight gain was included in the model. However, the association was significant only when we adjusted for concurrent exposure to di-*ortho* PCBs. Consequently, our results strongly suggest that it is important to control for concurrent exposure to different contaminants, such as PCBs and PBDEs, in studies of POPs and birth weight. Including several pollutants in the same model may however introduce over-adjustment bias if the pollutants are strongly correlated. In our study, the correlation between levels of di-*ortho* PCBs and PBDE(4) was weak (Pearson correlation coefficient 0.19) and collinearity did not bias the associations. The weak correlation between di-*ortho* PCBs and PBDE(4) is probably due to differences in exposure pathways. Food is the main source of human exposure to di-*ortho* PCBs, while both diet and indoor dust may be important sources of PBDEs [61].

Possible mechanisms responsible for the observed negative association between PBDE(4) exposure and birth weight are unknown but could involve effects on endocrine systems. For example, animal as well as epidemiological studies have shown that PBDEs can alter thyroid hormone levels [62-66], which may influence fetal growth.

Exposure to di-*ortho* PCBs as well as PBDEs may be a proxy for fish consumption since fish is the major contributor to the dietary intake of these substances in the Swedish population [16]. Maternal seafood consumption has been positively associated with birth weight in several observational studies [19]. Long-chain omega-3 fatty acids, abundant in fat from fish, may be responsible for this association, either by enhancing fetal growth or prolonging gestation [17,18,67]. In our study, the positive association between di-*ortho* PCBs exposure and birth weight was not appreciably changed when fish consumption during pregnancy was added to the regression model, thus indicating that omega-3 fatty acids in fish are not responsible for the observed association for di-*ortho* PCB. However, the inverse association between PBDE(4) and birth weight became stronger when fish consumption was added to the regression model. Fish consumption may consequently confound this association because of its positive association with both PBDE exposure and birth weight.

When female and male infants were studied separately, the observed associations were only significant in males (Table 4). The observed sex difference may be due chance or to a lower statistical power in the analyses of females. However, it is also possible that male fetuses are more sensitive to exposure to PCBs/PBDEs than females. Other studies have shown inverse associations between PCBs (or POPs in general) and birth weight restricted to male infants [20,21,38,68]. A lower sex ratio (increased probability of female births) among infants with fathers who were highly exposed to dioxins after the Seveso accident in 1976 [69] also indicates a higher susceptibility to POP among male fetuses. Sex-differences in effects of endocrine disrupting chemicals are commonly reported.

An important strength of our study is the exposure assessment including chemical analysis of both di-*ortho* PCBs and PBDEs. This made it possible to adjust associations for concurrent exposure. The study is also relatively large in comparison to previously published studies, although statistical power is a problem in the analyses of e.g. sex differences. The long recruitment period could have influenced the results since the breast milk levels of PCBs and PBDEs have decreased in the POPUP cohort over the years [24]. However, sampling year was not related to birth weight. Moreover, including sampling year as a covariate in the multivariate models did not change the associations. If any, the association for di-*ortho* PCBs became stronger (β changed from 143 to 189 in the multivariate analysis including gestational length). Available data on several life-style factors, including dietary habits, enabled us to evaluate several possible confounders. Some of the variables included in the final regression model were self-reported which adds uncertainty to the results, although a strong correlation between register and self-reported data was found for some variables. Data on gestational length was only available for 71% of the participants, and it is therefore difficult to conclude whether the observed effects on birth weight are related to gestational length (regression model without gestational length) or fetal growth (regression model including gestational length as covariate).

## Conclusions

Our relatively large study of background prenatal exposure to di-*ortho* PCBs and PBDE(4) indicates that these substances may influence birth weight in different directions, especially in boys. Exposure to di-*ortho* PCBs tended to be associated with higher birth weight while the association between PBDE(4) and birth weight was inverse. Importantly, we were able to adjust for maternal weight gain during pregnancy, a factor that should be consider in future studies since failure to control for this may result in false negative associations between di-*ortho* PCB exposure and birth weight. The observed associations may be causal, but it is also possible that some unmeasured or unknown confounding factors have influenced the results. The differences in birth weight associated with an increase in exposure from the 25th to the 75th percentile of exposure were relatively small (+100 and −80 g for di-*ortho* PCBs and PBDE(4) respectively). This can be compared with a difference in birth weight between non-smoking and smoking mothers of about 200 g [34,70]. Both high and low birth weight has been linked to e.g. overweight, hypertension and insulin resistance later in life [71]. We speculate that even small PCB- and PBDE-induced shifts in birth weight distribution may influence future public health in populations with background exposure, especially males in the lower or upper ends of the birth weight distribution.

## Abbreviations

ANOVA: Analysis of variance; BMI: Body mass index; LOD: Limit of detection; LOQ: Limit of quantification; NFA: Swedish National Food Agency; PBDE: Polybrominated diphenyl ether; PCB: Polychlorinated biphenyl; PCDD: Polychlorinated dibenzo-*p*-dioxin; PCDF: Polychlorinated dibenzofuran; POP: Persistent organic pollutant; POPUP: Persistent Organic Pollutants in Uppsala Primiparas; SD: Standard deviation; SE: Standard error; PBDE(4): sum of BDE-47, BDE-99, BDE-100, BDE-153; TEQ: Toxic equivalent; VIF: Variance inflation factor.

## Competing interests

The authors declare that they have no competing interests.

## Authors’ contributions

All authors have read the final version of the manuscript and are in agreement that the work is ready for submission to a journal. SL took part in the planning of the study and the acquisition of data, conducted the statistical analyses and did most of the writing. AG planned and designed the study, helped interpreting the results and took part in the preparation and reviewing of the manuscript. MA was responsible for the chemical analyses, for the quality control of analytical results and for the reviewing of the parts of the manuscript involving chemical analyses. POD planned the study and helped interpreting results and critically revise the manuscript. AH helped interpreting the results in a toxicology context and revised the manuscript. SCL helped planning the statistical analyses, interpreting the results and revised the statistical parts of the manuscript.
